# Understanding influences on teachers’ uptake and use of behaviour management strategies within the STARS trial: process evaluation protocol for a randomised controlled trial

**DOI:** 10.1186/s12889-015-1486-y

**Published:** 2015-02-10

**Authors:** Lorraine Hansford, Siobhan Sharkey, Vanessa Edwards, Obioha Ukoumunne, Sarah Byford, Brahm Norwich, Stuart Logan, Tamsin Ford

**Affiliations:** Child Mental Health Research Group, University of Exeter Medical School, Veysey Building, Salmon Pool Lane, Exeter, EX2 4SG UK; Primary Care Group, Plymouth University Peninsula School of Medicine & Dentistry (PUPSMD) N32, ITTCC Building Davy Road Tamar Science Park, Derriford, Plymouth, PL6 88X UK; Collaboration for Leadership in Applied Health Research and Care for the South West Peninsula, University of Exeter Medical School, Veysey Building, Salmon Pool Lane, Exeter, EX2 4SG UK; Centre for the Economics of Mental and Physical Health, Institute of Psychiatry, Kings College London, De Crespigny Park, London, SE5 8AF UK; Graduate School of Education, University of Exeter, St Luke’s Campus, Exeter, EX1 2 LU UK

**Keywords:** Process evaluation, Public health trial, Classroom behaviour management, Qualitative methods, Protocol

## Abstract

**Background:**

The ‘Supporting Teachers And childRen in Schools’ (STARS) study is a cluster randomised controlled trial evaluating the Incredible Years Teacher Classroom Management (TCM) programme as a public health intervention. TCM is a 6 day training course delivered to groups of 8–12 teachers. The STARS trial will investigate whether TCM can improve children’s behaviour, attainment and wellbeing, reduce teachers’ stress and improve their self-efficacy. This protocol describes the methodology of the process evaluation embedded within the main trial, which aims to examine the uptake and implementation of TCM strategies within the classroom plus the wider school environment and improve the understanding of outcomes.

**Methods/design:**

The STARS trial will work with eighty teachers of children aged 4–9 years from eighty schools. Teachers will be randomised to attend the TCM course (intervention arm) or to “teach as normal” (control arm) and attend the course a year later. The process evaluation will use quantitative and qualitative approaches to assess fidelity to model, as well as explore headteachers’ and teachers’ experiences of TCM and investigate school factors that influence the translation of skills learnt to practice. Four of the eight groups of teachers (n = 40) will be invited to participate in focus groups within one month of completing the TCM course, and again a year later, while 45 of the 80 headteachers will be invited to take part in telephone interviews. Standardised checklists will be completed by group leaders and each training session will be videotaped to assess fidelity to model. Teachers will also complete standardised session evaluations.

**Discussion:**

This study will provide important information about whether the Teacher Classroom Management course influences child and teacher mental health and well-being in both the short and long term. The process evaluation will provide valuable insights into factors that may facilitate or impede any impact.

**Trial registration:**

The trial has been registered with ISCTRN (Controlled Trials Ltd) and assigned an ISRCTN number ISRCTN84130388. Date assigned: 15 May 2012.

## Background

Prevalence of antisocial behaviour in children and adolescents has increased in recent years [1,2] and poor socio-emotional adjustment in early childhood increases the risks of psychiatric disorder, risk taking behaviour, educational failure, and involvement in crime in both childhood and adulthood [1,2]. These children also incur substantial costs to both society and their families [3]. The impairment and societal costs of antisocial behaviour, however, occur across the population distribution rather than just among those with the highest level of problems [3].

For teachers, disruptive behaviour in the classroom is associated with higher stress levels and burnout, and lack of training to manage this behaviour has been highlighted [4-6]. Children in poorly managed classrooms may see that disruptive behaviour results in staff attention, while good behaviour may rarely be acknowledged.

Jennings and Greenberg have proposed a model that highlights links between teachers’ social and emotional competence and well-being and the maintenance of teacher-student relationships, effective classroom management and successful implementation of social and emotional learning programmes for children [7]. They call for increased research to improve teacher social and emotional competence to help improve learning outcomes for students.

This protocol describes the embedded process evaluation within a cluster randomised controlled trial (RCT) that will evaluate whether a teacher classroom management programme leads to improved social-emotional competence, behaviour and learning among children and reduced stress, burn out and improved professional self-efficacy among teachers. MRC guidance [8] emphasises that a thorough process evaluation is essential to understand implementation issues in trials of complex interventions [9].

### The intervention: the teacher classroom management programme

While there are programmes that target children [10], a recent systematic review identified only two interventions that have been tested in randomised trials more than once, and which focus on enhancing teachers’ skills [11]. One of these programmes is the Incredible Years (IY) Teacher Classroom Management (TCM) programme [12]. TCM draws on cognitive social learning theory, in particular: Patterson’s theories concerning how coercive cycles of interaction between adults and children reinforce unwanted behaviour patterns [13]; Bandura’s ideas about the importance of modelling and self-efficacy [14], and Piaget’s developmental interactive learning methods [15]. TCM is delivered to groups of ten teachers, and involves six whole-day sessions spread over six months. It is delivered in a collaborative style by trained and supervised ‘group leaders’ who encourage teachers to share their experience and expertise and to value that of others. TCM uses goal setting, reflective learning, video modelling, role play, rehearsal of novel management strategies, group discussion, support and problem solving, and cognitive and emotional self-regulation training.

There have been only three trials of TCM in isolation from other interventions [16-18]. In a small observational study in Wales, 23 teachers reported high levels of satisfaction with TCM. Direct observation revealed that teachers who had accessed TCM gave clearer instructions, allowed more time for compliance and their pupils were more compliant [17]. This study preceded an RCT involving 12 classes from 11 primary schools and 107 children aged 3–7 years [16]. Independent classroom observations showed a significant reduction in classroom off-task behaviour, teacher negatives to target children, target child negatives towards the teacher and target child off-task behaviour. However, there was no significant change in teacher behaviour towards the whole class (possibly because teachers were aware that target children were being observed), and children at low risk of conduct disorder showed no change in negative behaviour. Another trial took place in Ireland, and follow-up is on-going [18]. All trials suggest that TCM is sufficiently intense to change teachers’ behaviour, and may improve behaviour for some children, but not enough is known about universal classroom effects. None of the trials looked at whether benefits to children and teachers are sustained in the longer-term. Other studies involving TCM have either added additional coaching for teachers or children, and/or studied the parallel parent and child programmes with or without TCM [19-22]. All suggest that TCM is potentially effective but given the additional interventions it is impossible to estimate the impact of TCM alone as a public health intervention.

### The STARS trial

The TCM programme is being evaluated in the Supporting Teachers And childRen in Schools (STARS) cluster randomised controlled trial, with embedded process and economic evaluations.

**Figure 1:**
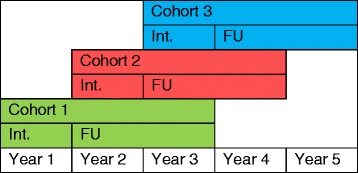
**Timeframe for STARS data collection.** Key: Int. – Intervention year. FU – Follow up years.

The setting is primary schools within Devon, Torbay and Plymouth, with one teacher and their pupils per school (cluster) allocated to TCM training or teaching as usual (TAU). Eighty schools will be recruited over three years in three overlapping cohorts; 15 in Cohort 1, 30 in Cohort 2 and 35 in Cohort 3. The trial design includes a one year pilot phase (Cohort 1), followed by a four year main phase (Cohorts 2 and 3). Each school will participate in the trial for three academic years, with a one year intervention period and two years of follow up (see Figure 1).

The duration of the intervention (six months) dictates that the TCM programme runs once per academic year between November and April. Teachers from schools allocated to the intervention arm will attend the course in their first year of participation and, as an incentive for participation, control teachers will attend in their second year of participation. By the end of the trial, 80 teachers will have attended TCM over a four year period, i.e. eight groups of 10 teachers.

The main outcome is teacher-reported child mental health, with secondary outcome measures of parent-reported child mental health, child behaviour, child academic attainment, child-reported enjoyment of school and teacher-reported mental health, relationship to work and sense of professional efficacy. Child and teacher outcomes will be assessed at the beginning and end of the first academic year (T0 and T1). Children will have new teachers in each follow-up year (T2 and T3), who will complete the child well-being measures. Child-reported outcomes will be measured in both follow-up years (T2 and T3). The study teachers will also be working with a new class of children, which allows us to offer the control teachers TCM in the second year of participation.

This protocol describes the qualitative and quantitative data to be collected in the process evaluation. For further information about the STARS trial, see Ford et al. [23].

### Theoretical basis to the STARS process evaluation

MRC guidance [8,24] suggests that a process evaluation can provide insight into why a successful intervention is working or why an intervention fails. Nested within a trial, process evaluation can increase understanding of trial quality and influences on implementation. However, Munro & Bloor [25] caution against collecting too much data in a process evaluation and highlight the need to prioritize both the type and amount of data collected. As well as pre-trial development, qualitative methods are advocated during and post-trial, to facilitate the interpretation of trial results [26,27]. One advantage of using qualitative methods during process evaluation is that they enable assessment of social processes during the implementation and uptake of an intervention, and also of the context within which implementation takes place [28].

The process evaluation will build on the earlier feasibility phase evaluation of the research processes from the schools’ perspective, to optimise the conduct of the trial. Also, to increase understanding of the personal and school-based practices and contexts which influence how TCM strategies are taken up, used and transferred between staff, the STARS process evaluation aims to develop a cohesive description of teacher and headteacher perspectives and experiences relating to their use of TCM. Accordingly, the aims of the process evaluation in the pilot and main phases reflect the different research questions of these stages of the trial.

### Process evaluation aims

#### Pilot phase: Year 1

*Aims*: To inform main trial processes, identify use of TCM strategies in the classroom and identify any additional sources of support.

*Research Questions:*

- How do teachers experience the TCM course content and delivery and what are the influences on experiences and uptake of learning?
- What are the teachers’ and headteachers’ views about the trial and research processes?
- How have teachers begun to use TCM strategies in the classroom and what influences this?
- Which additional sources of support are used by teachers in their management of challenging behaviours?

Both quantitative findings about attendance and course coverage, and qualitative findings about course content, delivery and trial processes will be summarised to identify aspects of the course that were more positively or negatively received than others in the delivery and content of TCM and how the trial is run. This information will be fed back to both the trial team and group leaders in time for any changes to be made prior to the commencement of the main phase.

### Main phase: Years 2-5

*Aims*: To examine TCM uptake and use in the classroom by teachers and other staff and influences on this, and to improve understanding of outcomes.

*Research Questions:*

*Within three months of completing the course*

- How do teachers experience learning and uptake of TCM strategies suggested by the course, and what influences this?
- How are teachers (and other staff) using TCM strategies in school and what influences this?
- What are the barriers and facilitators that influence the use of TCM strategies in the classroom?
- Which additional sources of support are used by teachers in their management of challenging behaviours?

*At one year following course*

- How do TCM strategies continue to be used, in what ways and what influences this?
- Which additional sources of support are used by teachers in their management of challenging behaviours over time?

Findings from the main phase will increase understanding about influences on teachers’ uptake of TCM strategies and the contextual influences on use of TCM strategies in classrooms. Findings will also illuminate how TCM strategies have been shared and disseminated within schools and any impact on the use of additional educational support. These findings will also support the interpretation of trial outcomes by providing contextual information about schools and teachers.

### Across both pilot and main phase

*Aims*: to record teacher attendance at TCM course and fidelity of TCM curriculum delivery.

*Research questions:*

- Are outcomes better for teachers who attend four or more sessions?
- Are some TCM sessions better attended than others?
- Is the TCM course being delivered with fidelity?

## Method

### Sample size and power

The STARS cluster RCT sample size was selected to provide 85% power at the 5% level of significance to detect a difference in the mean teacher Strengths and Difficulties Questionnaire (SDQ) score [29] between trial arms equivalent to an effect size of 0.3 of a standard deviation or a difference of 2 points on the raw SDQ scale [23]. The STARS trial will work with one headteacher, one teacher, their class and parents from each of 80 state funded primary schools. The class must be a single year group class between Reception and Year 4 (children aged between four to nine years at recruitment) and the teacher must be in the class at least four days per week. The target sample size of children at final follow-up is 1600.

This sample necessarily sets the sample frame for the process evaluation. As the control condition is to receive the intervention with a year’s delay, quantitative attendance and satisfaction data will be collected from all 80 teachers, and there will be eight courses, comprising six sessions each, conducted by six group leaders working in pairs. There is little methodological guidance when planning sample sizes for qualitative studies, but empirical study suggests that data saturation is possible within the first 6–12 interviews, although many more are often conducted as in the case in this study [30]. We will systematically review our data for data saturation and stop data collection if it is clear that data saturation has occurred, or conduct additional interviews/focus groups if new themes are still emerging at the end of the planned data collection.

Sampling within the qualitative part of the process evaluation will be purposive [30,31] to facilitate data collection of the views and experiences of the full range of participants who can comment on trial processes and the delivery and the uptake and use of TCM strategies. Focus groups with teachers will be carried out with four of the eight TCM groups from the three cohorts, and will include teachers from both intervention and control arms. Forty five headteachers will be invited to take part in a telephone interviews: 15 headteachers from Cohort 1 and the intervention headteachers from Cohorts 2 and 3. Fidelity and attendance data will be collected on all groups and from all teachers attending TCM.

### Data collection

#### Qualitative data

Data collection will run parallel to the pilot and main phases of the trial.

#### Focus groups

These are useful for collecting relevant and informative data on complex behaviour, such as what different groups think about a subject and why they hold such views [31]. Each focus group will have a researcher-facilitator and observer, will last for a maximum of one and half hours and will have ground rules (including confidentiality) and a clear structure. Researchers will follow a topic guide to facilitate discussion (available from the authors on request). There will be time for summary and reflection at the end of each group and participants will have an opportunity to discuss the focus group with a researcher via email should they wish. Intervention teachers unable to attend the focus group will be invited to take part in an individual telephone interview to elicit their views using the same topic guide.

#### Semi-structured interviews

Data will be collected using telephone interviews with headteachers. Telephone interviews can take the same form as face to face interviews, with a semi-structured format, but are usually shorter in length so can help improve the participation of busy professionals [32,33]. The interviews will last a maximum 30 minutes and will be pre-planned to optimise participation and privacy. The researcher will follow a topic guide (available from the authors on request).

Interviews and focus groups will be audio-recorded and transcribed for subsequent analysis.

### Pilot Phase (Year 1)

#### Course experience and research processes

The focus will be on teachers’ and headteachers’ experiences and views of the TCM course content and delivery and trial processes (recruitment, arrangements and data collection), aiming to identify any changes needed prior to main phase. All intervention group teachers (n = 10) will be invited to join a focus group which will run soon after the course finishes.

### Main Phase (Years 2–5)

#### Teacher learning and use of TCM strategies

In Year 2 two of the TCM groups (n =20) will be invited to attend focus groups*.* Additional sources of support used by staff to manage behaviour in the classroom will also be explored. In Year 3, one of the three TCM groups will be invited to join a focus group (n = 10) to provide additional data on perceptions of TCM and learning and use and transference of strategies. For teachers in their follow-up year (i.e. Years 2, 3 and 4) all teachers who attended a focus group the previous year will be invited to re-attend to explore the sustainability of teachers’ use of the TCM strategies.

#### Impact of course

In years 3 and 4 the headteachers from intervention schools (n = 30) will be interviewed one year post course to assess whether TCM has had any wider impact on their school, including any change in the use of outside support services, and dissemination within the school.

#### Quantitative measures

Data will be routinely collected relating to the administration of the TCM course to provide contextual information about the feasibility and acceptability of the course for teachers, and the fidelity with which groups are delivered.

#### Course attendance

The number of TCM sessions that each teacher attends out of a possible six will be recorded.

#### Teacher completed

Standardised session evaluations will be completed by teachers after each session that ask them to rate the content of the session, the videoed examples, the group leaders management of the session and the group discussions on a four point scale (“not helpful”, “neutral”, “helpful” and “very helpful”). Teachers will be encouraged to write any additional comments they may wish to on the feedback form. The Teacher Satisfaction Questionnaire will be completed after the final session to record the teachers’ view of and application of the techniques covered in the course. The questionnaire asks them to rate the course as “helpful”, “somewhat helpful” or “unhelpful” in developing their classroom management skills, whether they would recommend the TCM course to other teachers (“would not”, “might” and “would strongly”) and to rate 24 specific techniques covered in the course on a five point scale (“not all useful”, “not very useful” “neutral”, “a little bit useful”, “very useful”). Teachers are provided space to comment on anything that they would change about the course and the TCM strategies that they use regularly. Comments from all teachers’ feedback will be examined prior to each focus group to ensure adequate discussion of particular issues of pertinence to particular groups.

#### Group leader completed

Group leaders will complete standard checklists after each session that indicate which parts of the expected curriculum were covered in terms of concepts and strategies (coded: yes/no).

#### Video films

TCM sessions will be filmed for supervision with the Incredible Years Foundation, the developers of the TCM course. The group leaders will select a section of video from each session to be discussed as part of the supervision process and will be supported to apply for formal accreditation.

### Analysis

Recent MRC guidance on process evaluation of complex interventions [24] suggests that theory driven approaches, which assess whether an intervention works by focusing on the assumptions about why it is thought to work, can help to illuminate contextual influences on uptake and utility of an intervention and variation in outcomes. As indicated earlier, TCM draws on a number of social learning theories relevant to the process evaluation that will explore teachers’ experiences of individual, social (group) and contextual influences on their own learning within the course, and their uptake and use of TCM strategies. One example is Bandura’s [34]model of self-efficacy, in which he suggests that a range of influences such as role play, coaching, past experience and ‘group’ learning and feedback, as well as individual emotional state, may influence self-efficacy, and in turn behaviour and performance. This and other theories can help guide analytical questions, in order to illuminate contextual and individual influences and to help identify what worked well or not so well for teachers within the context of TCM.

#### Qualitative data

All audio-taped qualitative data will be transcribed verbatim and anonymised. Data will be stored using Nvivo software [35] and will be password protected. Analysis will draw on understandings of social learning [14] and on subtle realist perspectives to help identify experiences as the lived ‘reality’ of participants [36]. The ways in which participants account for their experiences within the context of the trial and their own schools-based experiences [37] will be explored. Thematic analysis of interview and focus group data will be framed by research questions and will also allow for more inductive analysis whereby emergent themes are also identified. This approach will help explain the experience and views of teachers and headteachers.

In the analysis, ‘keyness’ of themes does not relate to the frequency of occurrence but to whether a theme captures information relevant to the research questions, in this case relating to a range of experiences and views about behaviours and contexts relevant to TCM strategies [38]. The Framework Approach [39] will be used to manage data and aid systematic analysis (description and summary of key themes, patterns and links in the data), allowing the researcher to move between levels of abstraction during analysis and between a theory driven and more inductive approach, whilst also displaying the relevant data sources. This approach will help maintain a focus on the process evaluation objectives for the different phases of the study. Data analysis will be carried out separately for each stage of the pilot and main trial phases and findings will be synthesized to provide feedback at appropriate stages within the trial.

#### Rigour and reliability

Two researchers will be supported by an experienced qualitative researcher who will provide interview training and on-going supervision during data collection and analysis. A number of methods will be adopted to enhance rigour during the study including: Purposive sampling to increase representation and transferability; multiple researchers; peer debriefing which allows for discussion and identification of themes and concepts to increase representation; recording of analytical discussions; checks for thematic saturation and consistency; use of the framework approach and an audit trail of analysis decisions to help minimise bias and track variability between researchers [40].

#### Quantitative data

Based on experience during our feasibility work [41], the distribution of attendance data is predicted to be highly negatively skewed, with most teachers attending most sessions, which will necessitate the use of non-parametric approaches to summarise and test them for differences between control and intervention arms.

Satisfaction with each session will be explored by reporting the proportions that endorse each response for each facet of the session (overall, videos, group leader, group discussion) by group and by session to look for systematic differences in how teachers experience the course. These can then be explored further in the data from focus groups and interviews. We will seek systematic differences between the teacher reported experience of each session across the whole trial, and between each of the eight groups that will be run using chi-squared. Finally, we will summarise satisfaction at the end of the course by the proportion reporting that they found the course “very helpful” and that they would “strongly recommend it” to a colleague. Similarly, we will explore the responses on the five-point Likert scale in relation to the different teaching techniques to seek strategies that were particularly popular/unpopular and triangulate these results with themes that subsequently emerge from the focus groups and interviews.

Fidelity to course content will be assessed by reporting the proportion of the prescribed curriculum covered in each session and over all from the group leader checklists, and examining whether this increased over time as group leaders gained experience.

We will examine whether the effectiveness of the intervention for improving child behaviour outcomes (based on the teacher-reported Strengths and Difficulties Questionnaire) is greater for pupils of those teachers that attended more TCM sessions. Because teacher attendance of TCM sessions is likely to be associated with factors that impact on pupil outcomes we will account for this confounding using instrumental variable methods described by Dunn and Bentall [42].

### Ethics and consent

Ethical approval for this study has been obtained from the Peninsula College of Medicine and Dentistry Research Ethics Committee (now under the auspices of the University of Exeter Medical School Research Ethics Committee), reference Mar12/05b/141.

The STARS study is likely to elicit sensitive and confidential data and attention to ethics and participant confidence in and acceptance of researchers is crucial. Information about the process evaluation will be included in trial information sheets, and consent for participation in focus groups or individual interviews will be obtained at the same time as consent to take part in the trial. A reminder and additional information on focus groups will be given to teachers during the TCM course. Verbal consent will be taken for participation at the beginning of each interview or focus group. It will be made clear that participants have the right to withdraw their personal data from the study at any time.

## Discussion

The process evaluation will provide valuable outputs relevant to the aims and different phases of the STARS trial. Summary and illustrative data will be available for each phase of the trial, to facilitate further interpretation and evaluation of the TCM course, the ways in which teachers learn about and use TCM strategies and any findings on teacher and headteacher views about impacts of the course for children and schools.

Towards the end of the main phase data will be synthesised to facilitate fuller understanding and key messages about influences on how TCM is used and shared with colleagues. A final report will combine findings from across each of the trial phases, which will be analysed in tandem with the outcome measures of the STARS trial.

The process evaluation will also contribute to methodological considerations relevant to the use of qualitative research methods in public health trials.

### Trial status

As at time of submission data collection is on-going and the trial is in the main phase.

## Abbreviations

MRC: Medical Research Council
TCM: The Incredible Years Teacher Classroom Management course
STARS: Supporting Teachers and childRen in Schools trial
